# Impact of Smartphone Usage on Sleep in Adolescents: A Clinically Oriented Review

**DOI:** 10.7759/cureus.76973

**Published:** 2025-01-05

**Authors:** Nischal Krishna Macharla, Chandrasekar Palanichamy, Madhusudan Thirunarayanan, Mitthra Suresh, Arul Saravanan Ramachandran

**Affiliations:** 1 Psychiatry, Sri Ramaswamy Memorial Medical College Hospital and Research Centre, Sri Ramaswamy Memorial Institute of Science and Technology, Chengalpattu, IND; 2 Conservative Dentistry and Endodontics, Sri Ramaswamy Memorial Kattankulathur Dental College and Hospital, Sri Ramaswamy Memorial Institute of Science and Technology, Chengalpattu, IND

**Keywords:** adolescents, learning, psychiatry & mental health, sleep pattern, smartphone usage

## Abstract

Smartphones are an integral part of our lives due to benefits, such as easy accessibility to information, social connectivity, workplace/financial applications, and entertainment including games. The many benefits of a smartphone, like connectivity, increased productivity, availability of information, and portability are however balanced by negative health consequences. Excessive screen time can have multiple negative consequences, the most important of which are reduced time spent in socialization and sleep. It would be especially useful to know the impact of screen time on sleep from a bio-psycho-social perspective. Sleep plays a crucial role in memory and is essential for all stages of learning. Restorative sleep may also prepare the brain for new learning. The use of smartphones could disrupt lifestyle, sleep, and potentially, multiple cellular processes through their impact on circadian rhythms and lead to significant and widespread morbidity and mortality. Adolescents may be particularly at risk as they are found to be, in addition, watching screens of gaming consoles, TVs, and computers. These findings must be seen against the background of widespread sleep curtailment due to smartphone use and psychosocial stressors, especially in the learning populations (students and trainees). Epidemiological studies have consistently demonstrated the relationships between sleep and academic performance. The implications of circadian disruption for learning populations, such as university students and professionals training for careers in medicine, and other critical occupations, cannot be overstated. This review highlights the possible negative consequences of smartphone use on overall sleep health while emphasizing the importance of studying its impact, on mental health and learning outcomes, in different populations. The review aimed to explore the literature on the impact of smartphone use on sleep in young people and the consequences for their academic achievement. The method used was a comprehensive search strategy using Medline, PubMed, and Google Scholar databases with appropriate keywords and Boolean operators.

## Introduction and background

Mobile phones are mass-produced electronic devices that are an integral part of our lives due to benefits, such as easy accessibility to information, social connectivity, workplace/financial applications, and entertainment, including games. They are popular, especially because they provide these benefits instantly, due to their portability and size [1].

Smartphones, on the other hand, are technologically more advanced mobile devices, which can perform many of the functions of a computer, in addition to those of mobile phones, while having the same convenience and form factor. Given the multifaceted applications of a smartphone, it has also progressively become an intrinsic part of our lives and relationships. 

The many benefits of a smartphone like connectivity, increased productivity, availability of information, and portability are, however, balanced by negative health consequences, including neck pain, accidents, poor mental health, and sleep disturbances [2]. Adolescents may be particularly at risk as they are found to be, in addition, watching screens of gaming consoles, TVs, and computers. A systematic review of Indian adolescents revealed that smartphone addiction among adolescents was 39% to 44% on average. Nearly 300 million of the Indian population uses smartphones [3]. Surveys suggest that up to 50% of teens and 27% of their parents feel that they may be addicted to mobile phones [4].

These negative health consequences are repeatedly highlighted by the popular press. However, it must be kept in mind that many of these negative health consequences of smartphone use are speculative and alarmist. Mental health professionals need to have actionable evidence to counsel their clients in a balanced and appropriate manner based on available scientific evidence [5-6]. 

The objective of this clinically oriented comprehensive review was to explore the literature on the impact of smartphone use on sleep in young people and its consequences on their academic achievement. The methodology used was a comprehensive search strategy in Medline, PubMed, and Google Scholar databases using appropriate keywords and Boolean operators.

## Review

Methodology

To conduct this review, a comprehensive search was undertaken using Medline, PubMed, and Google Scholar. Keywords include “adolescents”, “psychiatry and mental health”, “learning”, “sleep pattern”, and "smartphone usage”. The search results were supplemented by reviewing the bibliographies of the articles found, ensuring a thorough exploration of the subject matter related to the influence of smartphone usage on sleep patterns, mental health, and the academic performance of adolescents. 

Negative aspects of widespread smartphone use

People using smartphones have problems structuring their daily routines due to the disruptive impact of notifications and screen time. When it comes to students, sleep disturbances may in addition interfere with their ability to reach their classrooms, pay attention, and ability to focus and learn [7]. The result is an inefficient lifestyle with subpar academic outcomes, leading to stress and poor mental health. Excessive screen time can have multiple negative consequences, the most important of which are reduced time spent in socialization and sleep. It would be especially useful to know the impact of screen time on sleep from a bio-psycho-social perspective. Many hypotheses have been put forward to explain the impact of smartphone use on sleep. First, the bright light emitted suppresses the secretion of melatonin reducing sleep efficiency, delaying sleep onset, and disrupting circadian rhythms [7].

Second, sleep displacement occurs, when the smartphone is used as an unstructured leisure time activity with no predefined beginning and end point, unlike real-world games and other entertainment activities like attending theatrical performances, movies, and arcades [8].

Third, sleep can be affected by the content consumed through the smartphone such as activating music, arousing sexual or violent images, and video games requiring hyper-alertness to anticipate the required responses. Not surprisingly, studies have found that mobile phone usage is associated with short sleep duration, reduced sleep quality, and daytime sleepiness. There is also some evidence to suggest that this relationship may be bidirectional, especially in adults where insomnia may be the cause and not the outcome of mobile use [8].

The Millennium Cohort Survey containing 11,553 responses suggested later bedtime and shorter nighttime sleep duration are associated with hyperactivity and inattention. Indeed sleep deprivation was found to exert a modulatory effect on the onset of psychiatric conditions, including emotional regulation and depression in adolescents [9].

In particular, bedtime smartphone use is predictive of later rising time, longer sleep latency, and worse sleep efficiency. The screen light emitted by mobile phones is of short wavelength and towards the blue length of the light spectrum, which is likely to delay sleep onset for a long duration after exposure. Therefore, screen time guidelines have been evolved for the various age groups specifically affected by smartphone addiction [10]. According to the Australian Department of Health (ADH), the Canadian Pediatric Society (CPS), and the American Academy of Pediatrics (AAP), the recommended screen time for adolescents should not exceed more than two hours per day [11]. 

Biological aspects

In mammals, multiple physiological, endocrine, behavioral, and molecular rhythms are governed by an endogenous clock in the suprachiasmatic nucleus (SCN) of the hypothalamus. It runs freely to a cycle of around 40 minutes and, therefore, needs to be re-synchronized with 24 hours per day. To reset this clock and synchronize it with the solar day, the SCN is entrained daily, by the dark-light cycle to which the organism is exposed [12].

A phase shift in the circadian rhythms of the SCN results in alterations of both the bedtime and wake-up time of an individual which moves to a time earlier in the day (phase advance) or later in the day (phase delay). Light exposure suppresses the melatonin secretion and resets the phase of the SCN, which depends on the timing, intensity, wavelength, duration, and previous exposures to light or darkness. Light exposure, early in the biological night induces a phase delay while exposure late in the night and towards the early morning elicits a phase advance. Notably, these responses are present in blind people also, since the photoreceptors wired for the circadian entrainment are distinct from the rods and the cones of the visual system. These circadian photoreceptors have their peak sensitivity to a light wavelength in the range of 446 to 477 nm (visible short wavelength). Thus, a short wavelength approximately of 460 nm blue light exposure at night evokes a greater phase shift, thus alerting the responses and causing melatonin suppression when compared to other wavelength light in the visual range of 380 to 700 nm. Evidence indicates that these spectrally targeted blue light sources are more efficient in evoking circadian changes such as alertness, faster reaction time, attention lapses, and changes in the electroencephalogram (EEG) while measuring the electrical activity of the brain [12].

Circadian photoreceptors in the retina use melanopsin as photopigment. Along with these receptors, there are specialized ganglion cells that project directly to the SCN. Human circadian rhythms like those of other diurnal species are sensitive to light throughout the biological day. Indeed there is no part of the biological day where light exposure does not affect the circadian rhythms. However, there is a phase delay period in the early biological night and a phase advance region in the late biological night with the transition region occurring late in the biological night. These shifts are evident even with light exposure for a couple of days in a week [12]. 

The human circadian system is sensitive to even low-intensity light especially if it follows many hours of darkness. Most studies show that modern humans get relatively little bright light exposure and spend most of the day in indoor light with an intensity of 200 lux. Such individuals become sensitive to even modest light intensity of 1,000 lux. In addition, even brief flashes of light can be incorporated into the human circadian rhythm if they occur after dark periods. Supplementary to the regular sleep times with darkness during the day, studies have shown that intermittent exposure to bright light throughout the night causes the aligning of the circadian rhythms with the nightshift work schedules [13,14].

The physiological functions of human beings exhibit approximately a 24-hour rhythm. In 2017, the Nobel Prize in Medicine was awarded for the molecular mechanism regulating these rhythms. This molecular mechanism comprises genes that increase the transcription of other genes, whose products, in turn, exert a negative feedback, on their gene expression. This autonomous transcription-translation feedback loop is present in every cell of the body. These feedback loops affect many genes that sub-serve the functioning and the timing of cell biology. These individual cellular clocks are kept in synchrony by the neural network that connects them to the central circadian clock of the organism [13,14].

In addition to this genetic mechanism, there have been circadian rhythms noted even in non-nucleated cells such as red blood cells. These non-genetic mechanisms could interact with the gene transcription-translation loops in unknown ways. These mechanisms point to the existence of the primal circadian clock that could be common to all species. The ability of visible light to synchronize with the biological clock of the photosynthetic marine dinoflagellate (*Gonyaulax polyedra)* has been known since 1958 [13,14].

Human chronobiology

The SCN plays a critical role in regulating photoperiodic behavior and physiology such as sleep, wakefulness, alertness, feeding, hunting/ aggressions, seasonal reproduction, body temperature, and hibernation [15].

A photoreceptive net consisting of the retinal ganglion cells and their axons (containing melanopsin), communicates directly with the brain influencing the circadian rhythms and the pupillary size, through projection to the SCN and the olivary pretectal nuclei, respectively. This photoreceptive system (as opposed to the functionally distinct visual system) encodes and transmits information about the ambient light intensity. Through this system, gross environmental illumination is integrated into the circadian, neuroendocrine, and neural behavioral systems of the human nervous system. Blue light appears to be the strongest synchronizing agent responsible for resetting the circadian rhythm, to keep the biological rhythms and the psycho-behavioral rhythms in phase with the solar day [14]. In humans, bright blue light between 0500 and 1700 hours causes a phase advance, while exposure outside this range results in a phase delay. In humans, the altering effects of blue-enriched light (such as those emitted from phone screens) appear to be stronger in the evenings and during the nighttime compared to the morning hours. Bright, continuous, blue light resets the SCN pacemakers which synchronizes the rhythms of subsidiary cellular clocks which would otherwise run free and go out of phase with each other and out of synchronism with the solar day [15].

Smartphones equipped with light-emitting diodes (LEDs) emit visible blue light of short wavelength in the range of 446 to 484 nm. This screen light alters the circadian rhythms and also affects the ocular system: it reduces the melatonin hormone secretions (which is controlled by the SCN and the pineal gland) and decreases the core body temperature and the distal-proximal skin temperature (due to vasodilation of the distal blood vessels), leading to an increased level of sleepiness in the mornings and the evenings. Cortisol secretion by the pituitary gland is also influenced by the SCN. Normally there is a rapid increase in the blood cortisol level 30 to 60 minutes after awakening. One hour of exposure to mobile screen light of wavelength (414 to 800 lux) in dim light alters the cortisol awakening response. About 30 minutes of exposure to smartphone screen light with a frequency range of 0.50-3.99 Hz also reduces the electroencephalographic (EEG) activity of the brain, leading to mental disturbances, reduced sleep quality, and increased anxiety [16].

These findings have led to the effective use of light therapy for seasonal affective disorder, premenstrual depression, bulimia, and circadian rhythm disorders related to sleep and dementia [17].

Public health importance

Sleep plays a crucial role in memory symbolization and integration, as noticed by researchers over a century ago. Recent advances in functional neuroimaging have revealed several compelling findings. These findings indicate that sleep is essential for all the stages of learning. Sleep provides the ideal environment for neural network-level changes in memory representation and stabilizes the brain for long-term retention. Restorative sleep also prepares the brain for new learning [18]. 

These findings must be seen against the background of the widespread sleep curtailment due to smartphone usage and psychosocial stressors, especially in the learning populations (students and trainees). Epidemiological studies have consistently demonstrated the relationships between sleep and academic performance [16]. Although no causative links have been shown to date, the International Agency for Research has identified some shift schedules that disrupt the circadian rhythms which serve as a potential carcinogen [19]. Melatonin suppression is also shown to be associated with diabetes mellitus type II [19].

A comparable study conducted in Japan aimed at evaluating the effects of mobile phone usage on sleep in adolescents. The results found that the increased phone use after turning off the lights before bed was linked to shorter sleep duration, poor sleep quality, insomnia, and greater daytime sleepiness [20]. This research highlighted the negative consequences of nighttime phone use on the overall sleep health of adolescents, emphasizing the importance of restricting screen time before sleep.

Multiple recent reviews found that adolescent mental health was significantly impacted by smartphone use. Chronic sleep deprivation leading to reduced cognitive ability/ school performance, social isolation, and self-harm was identified by multiple cross-sectional, longitudinal, and empirical studies in this population [21-23]. Associated comorbidities such as depression, anxiety, obsessive-compulsive disorder (OCD), attention-deficit/hyperactivity disorder (ADHD), and excessive alcohol intake were also identified in this population [24,25].

## Conclusions

The review was able to flag critical and clinically relevant concerns of public health importance related to the continuous usage of smartphones. Research has proved that the use of smartphones could disrupt circadian rhythms leading to disturbances in lifestyle, sleep, and multiple cellular biological processes.

It is mandatory to impose screen time restrictions and document the levels of impairment related to sleep patterns, negative mood swings, and memory loss produced in the learning population and adolescents. Counseling students about the detrimental consequences of smartphone use before bedtime and the significance of getting sufficient sleep for the achievement of academic success is essential. Some researchers have already found that restriction of mobile phone use for 30 minutes before bedtime has a greater potential for improving sleep, overall mental health, and academic performance of students.

The clinical importance of evaluating smartphone usage patterns in the field of mental health and de-addiction is also highlighted in this clinically oriented review. Despite its many potential benefits, such as social connectivity, educational tools, and financial applications, smartphone addiction has been shown to negatively impact the overall health of adolescents.
